# mCSM–NA: predicting the effects of mutations on protein–nucleic acids interactions

**DOI:** 10.1093/nar/gkx236

**Published:** 2017-04-04

**Authors:** Douglas E.V. Pires, David B. Ascher

**Affiliations:** 1 Centro de Pesquisas René Rachou, Fundação Oswaldo Cruz, Brazil; 2 Department of Biochemistry, University of Cambridge, Cambridge, UK; 3 Department of Biochemistry and Molecular Biology, University of Melbourne, Melbourne, Australia

## Abstract

Over the past two decades, several computational methods have been proposed to predict how missense mutations can affect protein structure and function, either by altering protein stability or interactions with its partners, shedding light into potential molecular mechanisms giving rise to different phenotypes. Effectively and efficiently predicting consequences of mutations on protein–nucleic acid interactions, however, remained until recently a great and unmet challenge. Here we report an updated webserver for mCSM–NA, the only scalable method we are aware of capable of quantitatively predicting the effects of mutations in protein coding regions on nucleic acid binding affinities. We have significantly enhanced the original method by including a pharmacophore modelling and information of nucleic acid properties into our graph-based signatures, considering the reverse mutation and by using a refined, more reliable data set, based on a new release of the ProNIT database, which has significantly improved the reliability and applicability of the methodology. Our new predictive model was capable of achieving a correlation coefficient of up to 0.70 on cross-validation and 0.68 on blind-tests, outperforming its previous version. The server is freely available via a user-friendly web interface at: http://structure.bioc.cam.ac.uk/mcsm_na.

## INTRODUCTION

The interaction of proteins with DNA and RNA is essential for a wide variety of cellular processes, in particular for the proper regulation of gene expression, and DNA replication and repair. Mutations in these nucleic acid binding proteins lead to a range of diseases including cancer and SCID (1–5). With the advances in high-throughput sequencing, there has been a significant demand for approaches capable of detecting the consequences of novel mutations on the intricate regulatory balance of the cell. Traditional methods, while slow and laborious, have allowed for direct determination of the impact of these mutations. Increasingly genomic sequencing is being used to guide diagnosis and treatment options for cancers, but experimental approaches are proving inadequate for dealing with the vast amounts of data and variation not only between individuals, but also from the faster evolving cancer genomes.

Many efforts to computationally predict and model the effects of mutations on protein structure and function have started to help unravel the link between genotype and phenotype (6–16). Predicting the effects of mutations on altering protein–nucleic acid interactions, however, has been more intractable, with most approaches relying upon the use of force-fields, with limited success or scalability.

We have previously used the concept of graph-based signatures to model a broad range of molecular phenomena. This has included the effect of mutations on protein stability (17,18), and interactions with other proteins (18,19), small molecules (20–22) and metal ions (8). We also used these signatures to scalably look at, for the first time, the effects of mutations on protein–nucleic acid binding affinities (18). The complexity of nucleic acid chemistry and binding also hindered the development of methods to assess the effects on mutations, which is compounded by the other structural effects that a mutation might exert of protein structure, folding and interactions. This also limited the availability of high quality data, necessary to develop new methods.

By using a subset of high-quality data from the ProNIT database (version 2.0) (23), we have developed mCSM–NA using our graph-based signature concept, a method that provides a reliable, scalable way to predict and characterize the effect of a single point missense mutation on protein–nucleic acid binding.

## MATERIALS AND METHODS

### Data sets and validation

In order to assess the applicability of our mutational cut-off scanning matrices (mCSM) graph-based signatures in predicting the impact of mutations on protein–nucleic acid binding affinities, a dataset derived from ProNIT was used. ProNIT is a collection of experimental thermodynamic parameters for wild-type and mutant protein–nucleic acid complexes, including the change in binding affinity (or Gibbs free energy of folding; Δ*G*), linked to published experimental structures of the complexes. A total of 331 single-point mutations across 38 different complexes were considered, 258 of which reduced affinity of the protein for the nucleic acid. The experimental distribution of the changes in binding affinity across this dataset is shown in Figure S1 of Supplementary Materials. The datasets were further classified based upon the nature of the nucleic acid - whether they included double-stranded DNA (dsDNA; 222 single-point mutations across 28 complexes), single-stranded DNA (ssDNA; 42 single-point mutations across 6 complexes) or RNA (67 single-point mutations across five complexes).

The free energy (Δ*G*) of a system can be represented as a thermodynamic state function. Therefore, the ΔΔ*G* of a mutation from the wild type to mutant (ΔΔ*G*_wt→mut_) is approximately equal to the ΔΔG of the reversed mutation from mutant to wild type protein (ΔΔ*G*_mut→wt_). In order to prepare a more balanced dataset, to avoid the potential bias of the machine learning methods from the naturally skewed datasets of experimental observations (originally only 74 mutations increased protein–nucleic acid affinity), the mutant structures were modeled using Modeller (24) and incorporated into the training dataset, for a total of 662 single-point mutations.

mCSM–NA was trained under 10-fold cross validation (see Supplementary Methods). It was further evaluated using two separate independent blind test sets. The first validation set was comprised of 79 missense mutations affecting RNA binding from 14 different protein–RNA complexes. This dataset was derived from the work involving recognition of hotspots by Barik *et al*. (25). The second set includes four well characterized inactivating and rescue mutations on the tumor suppressor protein p53 (26). The structure of the p53–DNA complex (PDB: 4HJE) was used. Training and test sets, including the protein structures used, are available to download on the webserver at http://bleoberis.bioc.cam.ac.uk/mcsm_na/data.

### Graph-based structural signatures

mCSM–NA uses our well validated graph-based structural signatures to represent the protein–nucleic acid complex, that model both the geometry and physicochemical properties of the interactions and architecture of the wild-type structure. Our signatures represent atoms characterized as nodes and their interactions as edges, with their physicochemical properties encoded based upon the amino acid residue properties, denoted by a pharmacophore. In addition to our previous atom typings in the signatures modelling protein residue atoms, additional information was provided to encompass the nature of the nucleic acid bases. Two different classes of pharmacophores were evaluated. The first classifies nucleic acid atoms based on the nature of the nucleotide: atoms were labelled as belonging to purines or pyrimidines. The second modeling divides the nucleotide in phosphate, sugar and base, with atoms labeled based on the group they belong. The later pharmacophore modelling was the best performing option and was chosen.

**Figure 1.**
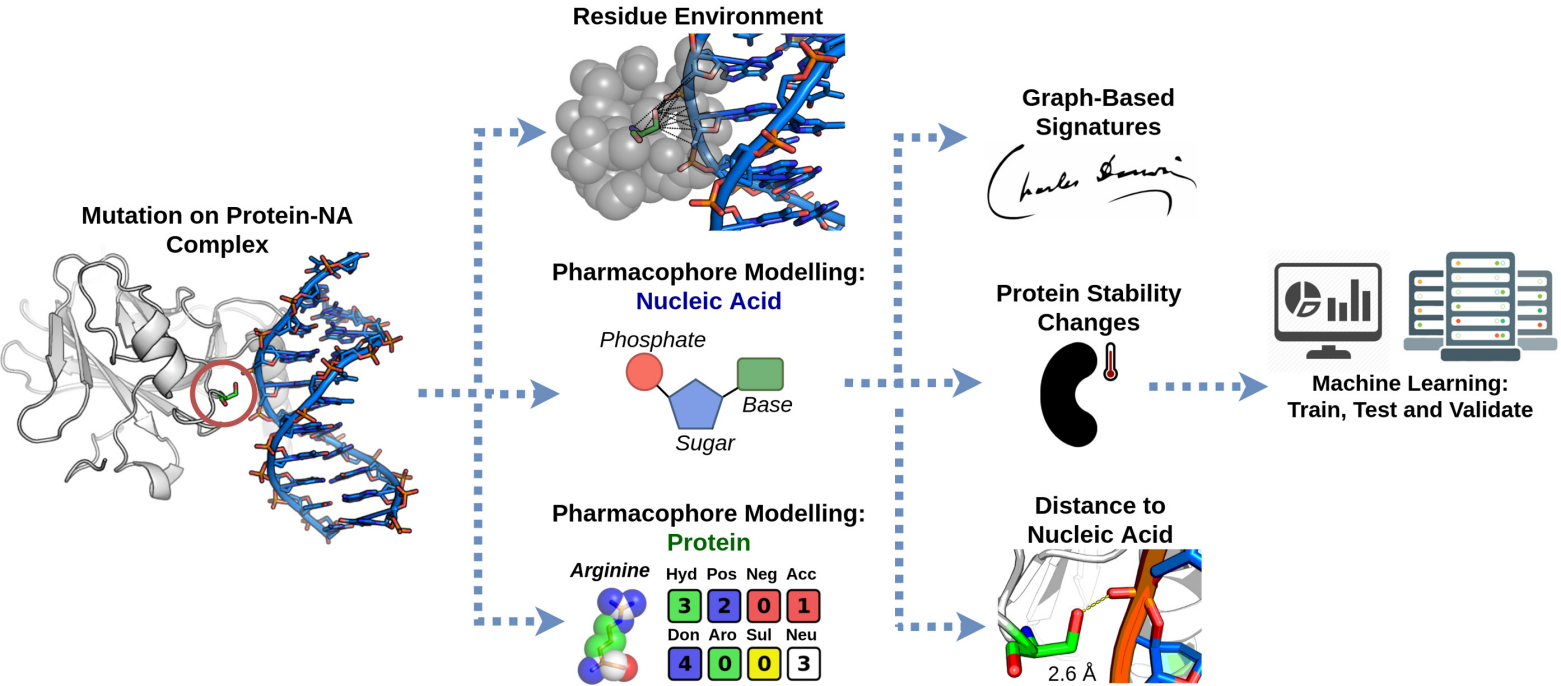
mCSM–NA workflow and application. The method relies on graph-based structural signatures that model distance patterns on the wild-type residue environment. Atoms at the vicinity of the mutated residue are labelled based on a pharmacophore modeling which are then used on the signatures to describe both geometry and physicochemical properties of the environment. Complementary information including distance from mutated residue to nucleic acid and predicted protein stability change upon mutation are also used to train, test and validate the predictive model.

From this representation of the residue environment, distance patterns between atoms characterized by their properties are summarized in concise signatures as cumulative distributions, and used as evidence for machine-learning methods. Complementary information including the distance between mutated residue to the nucleic acid and the predicted protein stability change upon mutation are also used as evidence to train and test the predictive models. Different supervised learning algorithms for regression implemented and available on the Weka Toolkit (27) were evaluated based on the Pearson's correlation coefficient and the best performing method selected (Gaussian Process) (28). The mCSM–NA prediction workflow is shown in Figure 1.

## WEBSERVER

We have implemented mCSM–NA via a user-friendly freely available webserver, available at: http://structure.bioc.cam.ac.uk/mcsm_na. The server front end was built using Bootstrap framework version 3.3.7, while the back-end was built in Python via the Flask framework (Version 0.10.1), on a Linux server running Apache.

### Input

The server provides two different input options for the user, as shown in the job submission interface (Supplementary Figure S2). The ‘Single Mutation’ option allows the user to predict the effect of a single mutation on the binding affinity of a protein–nucleic acid complex. The information required includes a PDB file or a PDB code of the protein–nucleic acid complex and nucleic acid type (either RNA, ssDNA or dsDNA), the point mutation specified as a string containing the single letter code of the wild-type residue in the protein, its corresponding residue number and the single letter code of the mutant residue, and the chain identifier of the residue. The ‘Mutation list’ option allows the user to upload an input file containing a list of up to 20 mutations and chain identifier (input string format similar to the first option) each in a separate line.

The predictions are performed as a regression task (numerical prediction of the difference in the Gibb's free energy of binding, ΔΔ*G*). In order to aid users to submit their jobs, sample submission entries are available on the submission page and a help page has been implemented and is accessible via the top navigation bar.

### Output

**Figure 2.**
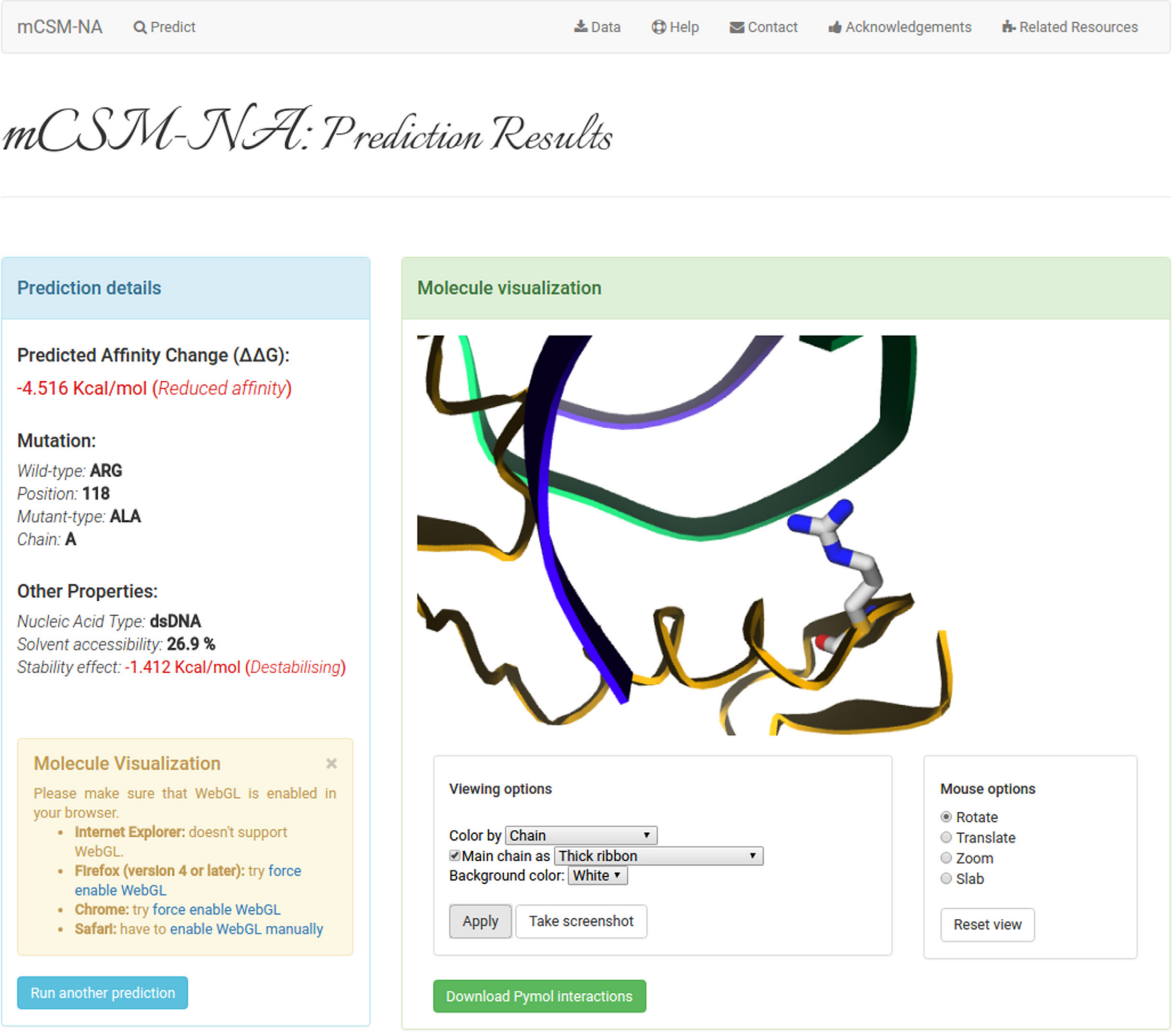
Web server results page for a single mutation prediction. The predicted change in affinity upon mutation (ΔΔ*G* in kcal/mol). Complementary information also displayed include nucleic acid type, residue solvent accessibility and predicted effect on protein stability. The protein complex and mutated residue can be visualized directly from the server, also allowing the users to download a pymol session of the residue and its interactions.

For the ‘Single Mutation’ option, the webserver outputs the predicted change in binding affinity in Kcal/mol, along with a summary of the input (Figure 2). A negative value (and red writing) corresponds to a mutation predicted as reducing affinity; whilst a positive sign (and blue writing) denotes a mutation predicted to increase the binding affinity.

A separate panel lists the structural features that include relative solvent accessibility and the predicted effect of the mutation on protein stability. The uploaded PDB file with its wild type residue environment can be visualized directly from the server using GLmol molecular viewer, and a Pymol session file showing all the intra- and inter-molecular interactions made by the wild type residue, calculated by Arpeggio (29), is available for download and viewing in Pymol for preparation of publication quality figures and to allow further analysis.

The results page for predicting the effects of a list of mutations is given in tabular format (Supplementary Figure S3). The identification of the mutation, its relative residue solvent accessibility, predicted change in binding affinity and protein stability (both as ΔΔ*G* in kcal/mol) are displayed. The results can be downloaded as a tab-separated file.

## VALIDATION

### Performance on cross validation

**Figure 3.**
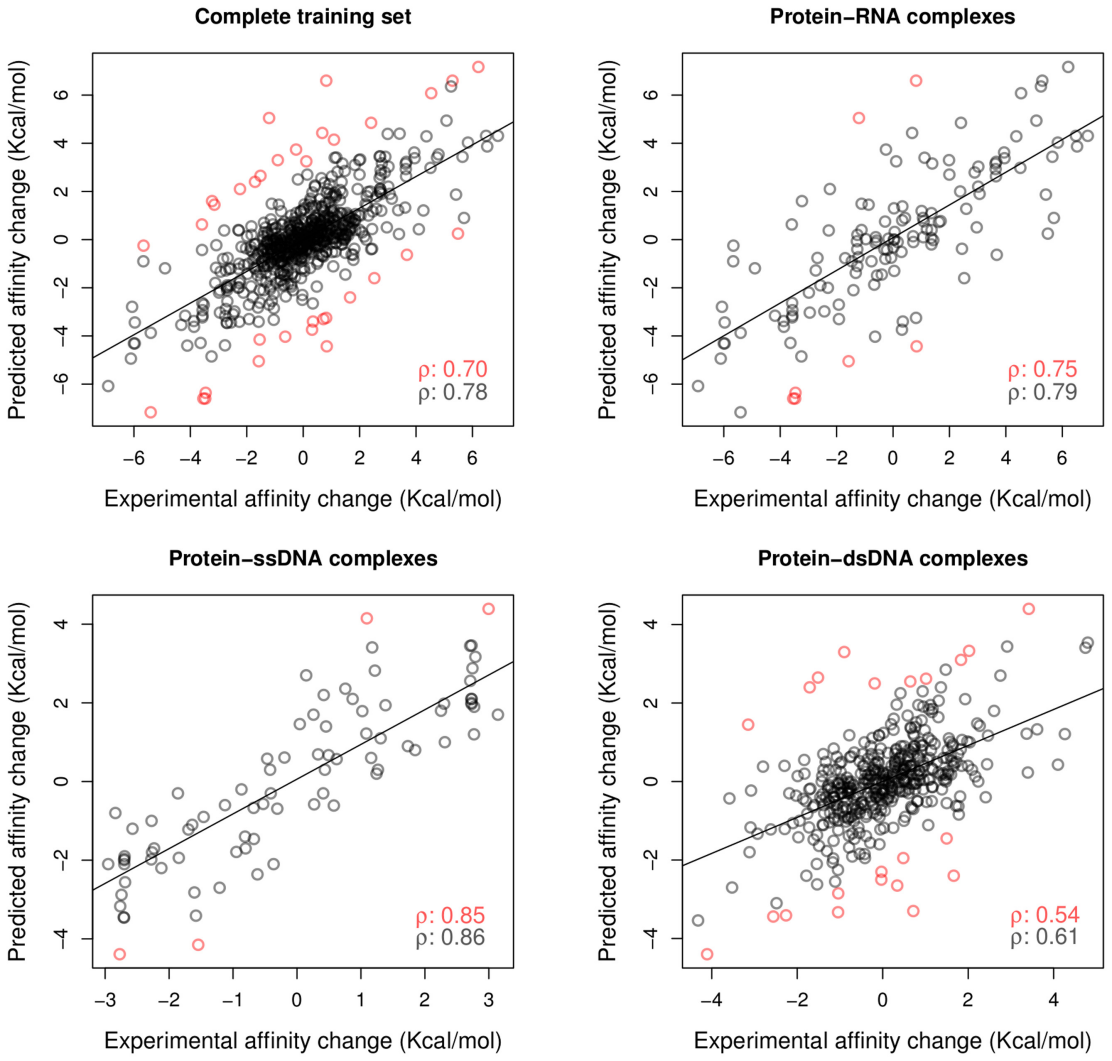
Regression plot between the experimental and predicted changes in binding affinity (in kcal/mol) during cross-validation. mCSM–NA obtained a Pearson's correlation of 0.7 across the original data set (**A**). The performance of the model against complexes containing RNA (**B**), ssDNA (**C**) and dsDNA (**D**) are shown, highlighting the accuracy and applicability of mCSM–NA to handle all different types of protein–nucleic acid complexes. The overall Pearson correlation coefficients, including outliers, is shown in red; with the correlation after removing outliers shown in black.

A series of experiments were carried out to assess the performance of mCSM–NA in predicting effects of single point missense mutations on protein–NA affinity for different nucleic acid types. Figure 3 depicts the regression plots between experimental and predicted mutation effects. For the complete training data set, mCSM–NA achieved a Pearson's correlation of *ρ* = 0.7 (top-left graph). After 10% outlier removal, the correlation increases to *ρ* = 0.78.

Across all types of nucleic acid interactions, mCSM–NA was able to accurately identify single point missense mutations leading to increased or decreased nucleic acid binding affinity (Supplementary Table S1). mCSM–NA performed better for mutations on complexes involving single-stranded nucleic acids (ssDNA and RNA) in comparison with dsDNA. For mutations on protein–RNA complexes (Figure 3, top-right graph), mCSM–NA achieves a Pearson's correlation of ρ = 0.75 and ρ = 0.85 for ssDNA (Figure 3, bottom-left graph), while achieving ρ = 0.54 for dsDNA (Figure 3, bottom-right graph), increasing to ρ = 0.61 after 10% outlier removal.

### Blind tests

In order to proper evaluate the generalization of the predictive model, two blind tests were carried out. On the first one, composed by 79 missense mutations on protein–RNA complexes, mCSM–NA achieve a Pearson's correlation of ρ = 0.56 (Supplementary Figure S4). The method, as expected, achieves a better correlation when assessing direct effects on nucleic acid affinity. When considering mutations within 10 Å of the nucleic acid the correlation between experimental and predicted effects increases to ρ = 0.63, while when only considering mutation directly in contact with the nucleic acid (within 5 Å) the correlation increases to ρ = 0.68.

On the second blind test, mCSM–NA was capable of differentiating between loss of function and respective rescue mutations on p53, showing a balanced performance between mutations increasing and decreasing nucleic acid affinity. The two loss of function mutations, R273C and R273H, were predicted to significantly reduce affinity between protein and nucleic (predicted ΔΔ*G*s of –1.220 and –1.352 kcal/mol, respectively), while the rescue mutations (T248R and S240R) were predicted to increase affinity (predicted ΔΔ*G*s of 0.430 and 0.198 kcal/mol, respectively).

## SUMMARY

We present here an updated version of mCSM–NA, which relies upon graph-based signatures to predict the impact of a single point missense mutation upon the nucleic acid binding affinity. mCSM–NA was trained on a newer and more reliable version of the ProNIT database also incorporating on its signatures nucleic acids pharmacophores as well as considering modeled reverse mutations to avoid biases between increasing and decreasing affinity during training. The method achieved a correlation of up to 0.7 during training and 0.68 on blind tests and was also able to differentiate between loss-of-function and rescue mutations on p53, showing its applicability in a real-world scenario. While there is still room for improvement in terms of performance on double-stranded nucleic acids, we believe mCSM–NA is an invaluable tool for mutation prioritization to guide experimentation, shedding light into the mechanistic effect of mutations on a molecular level through an intuitive and efficient web interface.

## AVAILABILITY

The server is freely available via a user-friendly web interface at: http://structure.bioc.cam.ac.uk/mcsm_na.

## Supplementary Material

Supplementary Data
