# Supplementary material for: mCSM–NA: predicting the effects of mutations on protein–nucleic acids interactions
Source: Nucleic Acids Res. 2017 Apr 4;45(Web Server issue):W241–6. doi: 10.1093/nar/gkx236 (PMC5570212; doi:10.1093/nar/gkx236)
Supplement: Supplementary Data [file gkx236_Supp.doc]

**Supplementary Materials**

**mCSM-NA: Predicting the effects of mutations on protein-nucleic acids interactions**

Douglas E.V. Pires^1^,*, David B. Ascher^1,,2,3^,*

^1^Centro de Pesquisas René Rachou, Fundação Oswaldo Cruz

^2^Department of Biochemistry, University of Cambridge;

^3^Department of Biochemistry and Molecular Biology, University of Melbourne.

*To whom correspondence should be addressed. D.B.A. Tel: +61 90354794; Email: [david.ascher@unimelb.edu.au](mailto:david.ascher@unimelb.edu.au) or [da382@cam.ac.uk](mailto:da382@cam.ac.uk); Correspondence may also be addressed to D.E.V.P. [douglas.pires@cpqrr.fiocruz.br](mailto:douglas.pires@cpqrr.fiocruz.br)


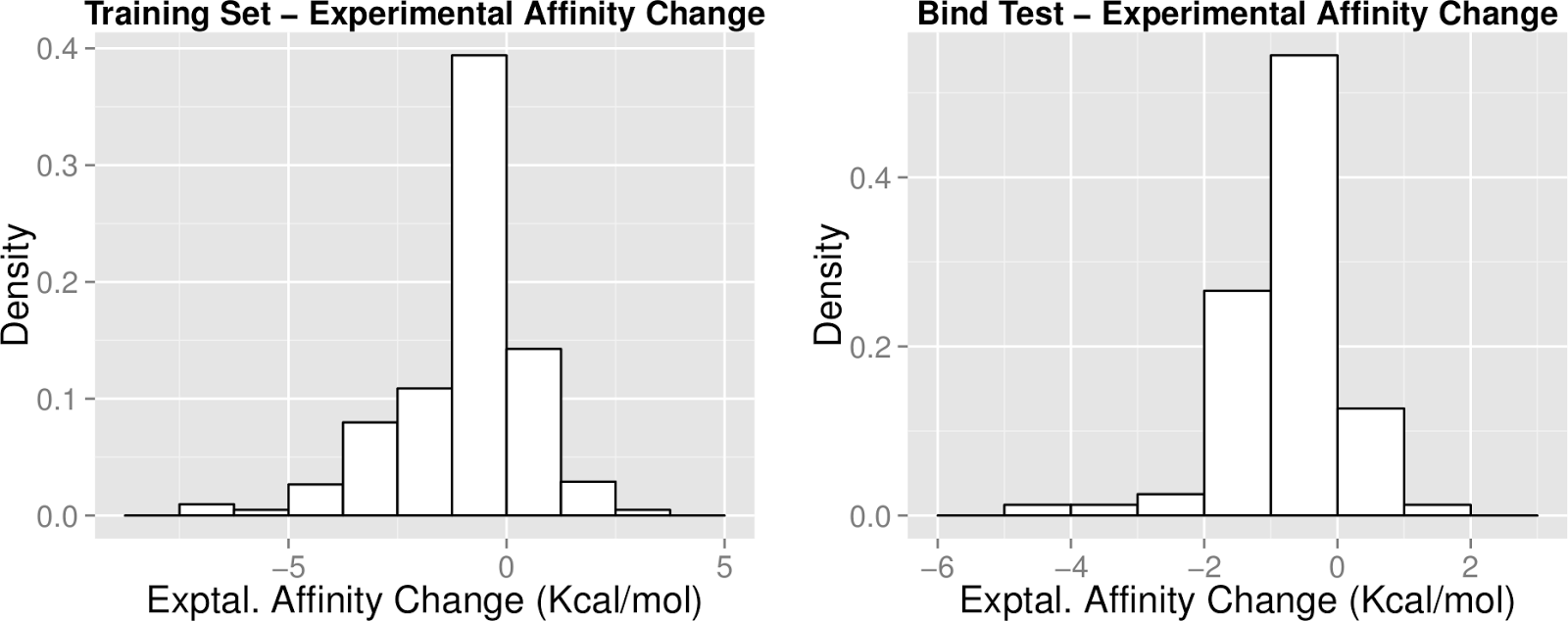


**Figure S1.** Histogram of experimental binding free energies.


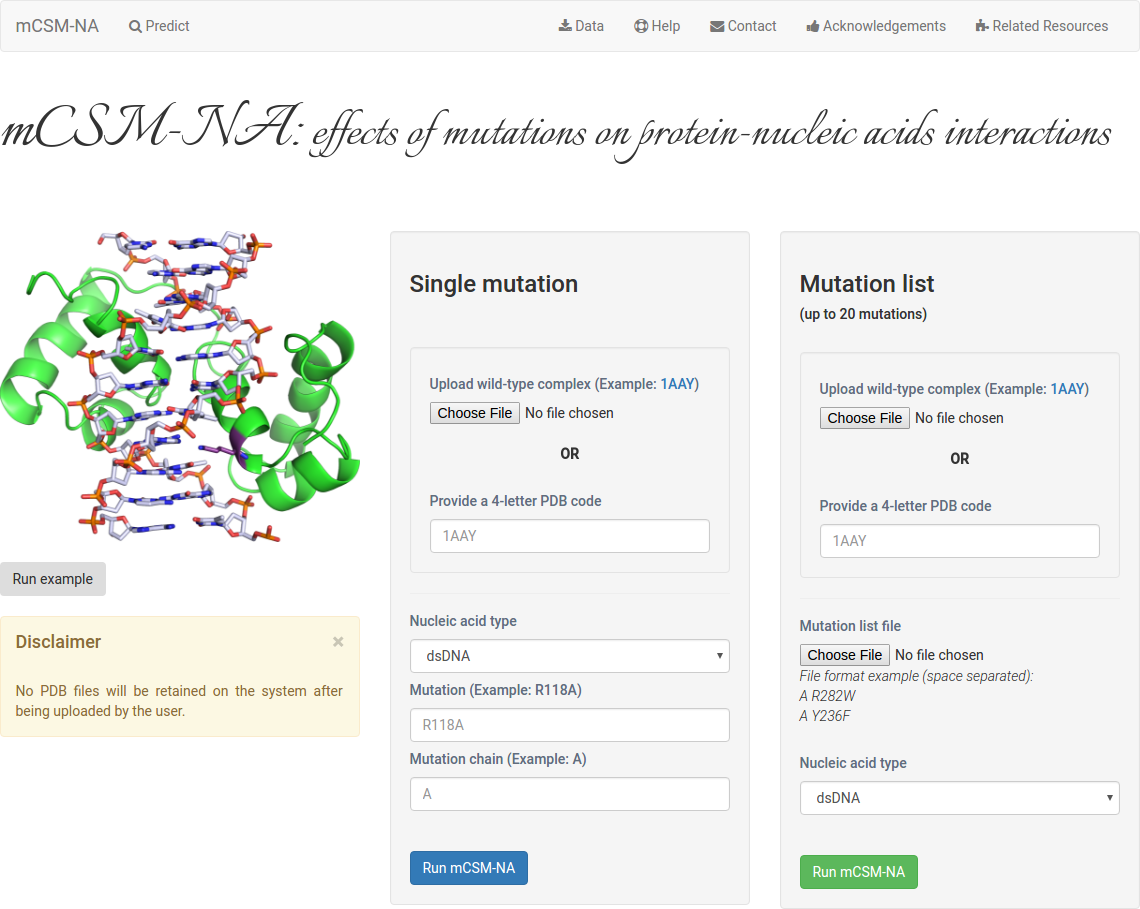


**Figure S2.** Job submission interface. Users can choose between submitting a single mutation or a list of mutation for analysis, as a separate file. The wild-type complex need to be uploaded and the nucleic acid type chosen between, RNA, single-stranded DNA (ssDNA) and double-stranded DNA (dsDNA).


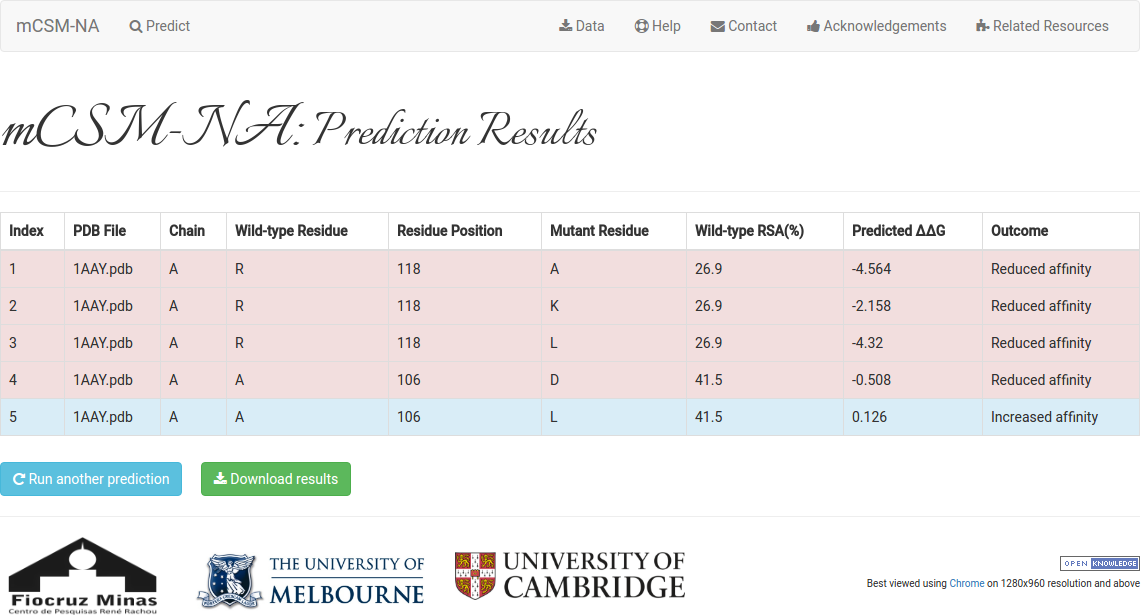


**Figure S3.** Results page for list of mutations. The web server will present the prediction results in a tabular form, which is also available for download as a tab-separated file.


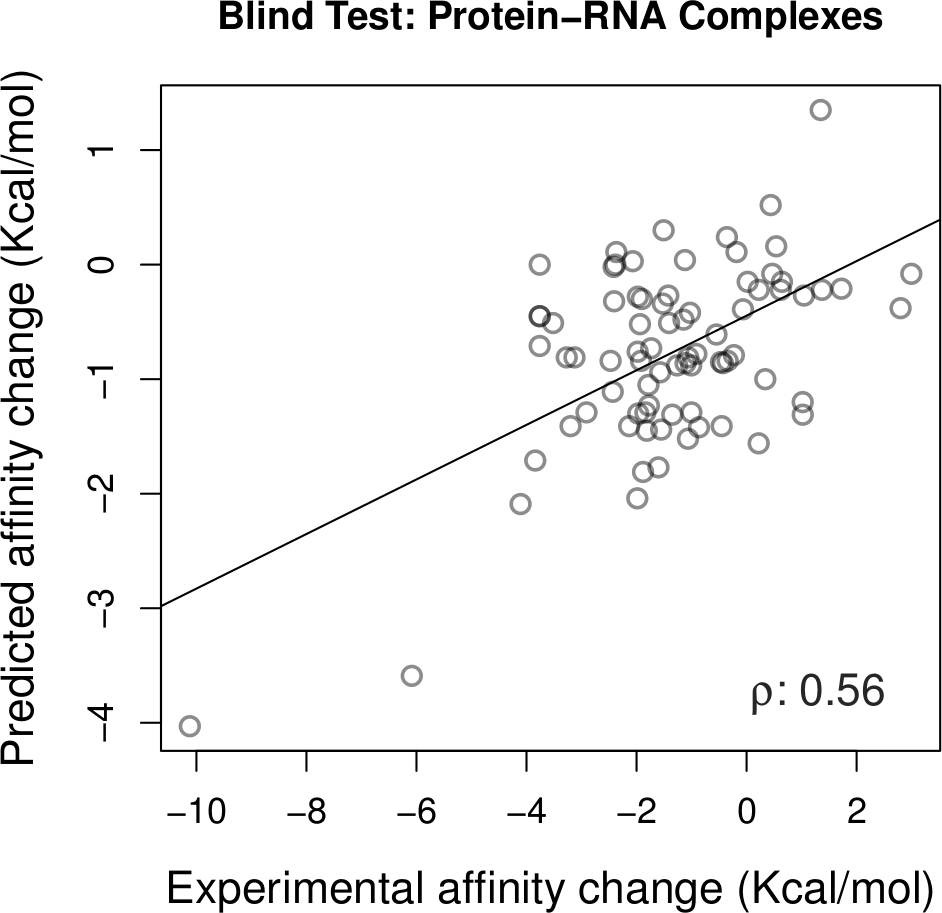
 **Figure S4.** Regression plot between the experimental and predicted changes in binding affinity (in Kcal/mol) during blind tests. mCSM-NA obtained a Pearson’s correlation of 0.56 across the original data set. When only selecting mutations within 10 Å of the nucleic acid, mCSM-NA achieved a correlation of 0.63 and 0.68 for mutations within 5 Å of the nucleic acid.

**Table S1.** Classification by regression performance of mCSM-NA during 10-fold cross validation.

| **Data set** | **Accuracy** | **Precision (Incrasing)** | **Precision (Decreasing)** |
| --- | --- | --- | --- |
| Full training set | 76% | 77% | 77% |
| Protein-RNA complexes | 78% | 79% | 79% |
| Protein-ssDNA complexes | 93% | 93% | 93% |
| Protein-dsDNA complexes | 72% | 74% | 73% |

# Supplementary Methods

### Machine Learning Approach

Different supervised learning algorithms for regression currently available on the Weka Toolkit, version 3.6.10, were evaluated under 10-fold cross validation. These included Gaussian Process, Regression Trees (M5P) and K-Nearest Neighbours (KNN). The best performing model was selected based on the Pearson's Correlation Coefficient across the training set.

• Gaussian Process Regression (Rasmussen and Williams, 2006):

A Gaussian Process *f(x)* is a collection of random variables that present a joint multivariate normal (Gaussian) distribution. It is specified by a mean function *m(x)* and a covariance (kernel) function *k(x, x )*:

*f(x) ∼ GP(m(x), k(x, x ))*

Gaussian Process Regression is a flexible supervised learning approach for regression and non-linear interpolation, which also gives uncertainty estimates of the predictions. In this work we used a RBF Kernel as covariance function (noise=0.5).
